# A Redox Amplification Interface Linking Mitochondrial Dysfunction, Immune-Derived Oxidants, and Biomaterial Electrochemistry in Chronic Inflammation

**DOI:** 10.3390/ijms27094121

**Published:** 2026-05-05

**Authors:** Żaneta Anna Mierzejewska, Bożena Antonowicz, Łukasz Woźniak, Jérôme R. Lechien, Luigi Angelo Vaira, Stanisław Dziełak, Jan Borys

**Affiliations:** 1 Institute of Biomedical Engineering, Faculty of Mechanical Department, Bialystok University of Technology, Wiejska 45C, 15-351 Bialystok, Poland; stanislaw.dzielak@pb.edu.pl; 2 Department of Dental Surgery, Medical University of Bialystok, M. Sklodowskiej-Curie 24A, 15-276 Bialystok, Poland; lukasz.wozniak@umb.edu.pl; 3 Department of Surgery, UMONS Research Institute for Health Sciences and Technology, University of Mons, Place du Parc 20, 7000 Mons, Belgium; jerome.lechien@umons.ac.be; 4 Department of Otolaryngology-Head & Neck Surgery, Foch Hospital, Paris Saclay University, 921500 Suresnes, France; 5 Maxillofacial Surgery Operative Unit, Department of Medicine, Surgery and Pharmacy, University of Sassari, Viale San Pietro 43B, 07100 Sassari, Italy; lavaira@uniss.it; 6 Department of Maxillofacial and Plastic Surgery, Medical University of Bialystok, M. Sklodowskiej-Curie 24A, 15-276 Bialystok, Poland; jan.borys@umb.edu.pl

**Keywords:** peri-implantitis, redox imbalance, mitochondrial dysfunction, oxidative stress, biomaterial corrosion, Nrf2

## Abstract

Peri-implant inflammatory disease exhibits marked clinical heterogeneity that cannot be explained solely by microbial burden, indicating the involvement of host-driven amplification mechanisms. This review integrates evidence from redox biology, immunometabolism, and biomaterials science to describe a redox amplification interface (RAI) linking immune-derived reactive oxygen species (ROS), mitochondrial dysfunction, and biomaterial electrochemical reactivity at the host–implant interface. Persistent NADPH oxidase activation promotes mitochondrial oxidative damage, including electron transport chain disruption, cardiolipin oxidation, and ROS-induced ROS release, resulting in sustained intracellular oxidative flux. Mitochondrial dysfunction further contributes to inflammatory amplification through release of damage-associated molecular patterns and activation of inflammasome signaling. Concurrent impairment of antioxidant systems, particularly Nrf2-dependent pathways and glutathione depletion, reduces redox buffering capacity and facilitates propagation of oxidative stress. Inflammatory microenvironments also destabilize implant surface electrochemistry, promoting corrosion, ion release, and surface-mediated redox reactions that increase local oxidative burden. These interacting processes form a coupled system capable of sustaining inflammation independently of the initiating microbial stimulus. This framework provides a mechanistic basis for disease heterogeneity and identifies redox-targeted therapeutic and biomaterial design strategies.

## 1. Introduction

Chronic inflammation at biomaterial interfaces cannot be explained solely by microbial burden, indicating the presence of intrinsic amplification mechanisms within host redox system inflammation [1,2,3]. Although microbial biofilms initiate host immune activation at the implant–tissue interface, comparable microbial burden frequently results in divergent outcomes, ranging from stable peri-implant conditions to rapid and progressive bone loss [4,5].

From a clinical perspective, peri-implantitis represents a prevalent and therapeutically challenging condition, affecting approximately 20–25% of patients and up to 10–15% of implants depending on diagnostic criteria [1,6,7]. Importantly, treatment outcomes remain unpredictable, with substantial variability in response to both non-surgical and surgical interventions [8,9,10].

In addition to redox-mediated mechanisms, peri-implant pathology may also be influenced by mechanical factors, such as micromotion or occlusal overload, as well as immunoallergic responses to biomaterials. These processes may contribute to inflammatory activation and tissue remodeling; however, they do not fully explain the persistence and amplification of inflammation. In this context, the redox amplification interface (RAI) framework provides an integrative perspective linking these stimuli with sustained oxidative signaling at the host–implant interface.

Recent advances in redox biology and immunometabolism have identified reactive oxygen species (ROS) as central regulators of immune activation, mitochondrial function, and skeletal remodeling. Under physiological conditions, ROS act as tightly controlled signaling mediators coordinating antimicrobial defense, angiogenesis, and osteogenic differentiation [11,12,13,14]. These processes are constrained by integrated antioxidant systems, most prominently the Nrf2-Keap1 pathway and glutathione-dependent buffering networks, which maintain redox homeostasis and prevent propagation of oxidative injury [15,16,17,18].

Within this altered redox landscape, mitochondria emerge as central amplification nodes that convert transient oxidative stimuli into sustained intracellular ROS flux. Oxidative damage promotes ROS-induced ROS release, a key mechanism underlying self-amplifying oxidative signaling [19,20]. In parallel, mitochondrial injury contributes to activation of inflammasome pathways and reinforcement of inflammatory responses [21,22]. Importantly, failure of antioxidant buffering systems, including impaired Nrf2 signaling and depletion of intracellular glutathione, lowers the threshold for oxidative propagation and sensitizes mitochondrial networks to dysfunction [15,16,17,18]. Together, these processes establish a self-reinforcing immune–mitochondrial feedback loop capable of sustaining inflammation independently of the initiating stimulus [19,20].

Biomaterial interfaces represent a unique redox-active environment in which implanted materials actively participate in shaping local oxidative dynamics [23,24]. Under inflammatory conditions, destabilization of protective oxide layers may promote corrosion, ion release, and surface-mediated redox reactions, introducing additional sources of oxidative stress that directly interact with immune signaling and mitochondrial function [24,25,26]. However, these processes are typically investigated in isolation, and a unifying framework integrating immune redox signaling, mitochondrial amplification, and biomaterial electrochemistry remains lacking [5,10].

A redox amplification interface framework is proposed, in which immune-derived ROS, mitochondrial dysfunction, and biomaterial electrochemical reactivity form a dynamically coupled system at the host–implant interface. Within this model, corrosion-driven redox processes, mitochondrial ROS amplification, impaired antioxidant buffering and material-driven oxidative inputs interact to establish a self-reinforcing pathological circuit that sustains inflammatory signaling and promotes tissue destruction. This framework provides a systems-level integration of intracellular and interface-level oxidative processes and generates experimentally testable predictions connecting mitochondrial dysfunction, oxidative biomarkers, and material-dependent electrochemical behavior.

The RAI model introduces several conceptual advances. It positions biomaterials as active redox modulators contributing to oxidative signaling networks, identifies mitochondria as central amplification nodes linking immune-derived ROS with sustained intracellular oxidative flux, and integrates extracellular electrochemical processes with intracellular redox signaling into a unified system. It also provides a mechanistic explanation for disease heterogeneity based on variability in host redox resilience and mitochondrial susceptibility.

Importantly, the RAI framework generates experimentally testable predictions linking oxidative biomarkers, mitochondrial dysfunction, and material-dependent corrosion processes, thereby enabling direct experimental validation and supporting a translational bridge between molecular redox mechanisms and clinically measurable oxidative phenotypes.

**Figure 1:**
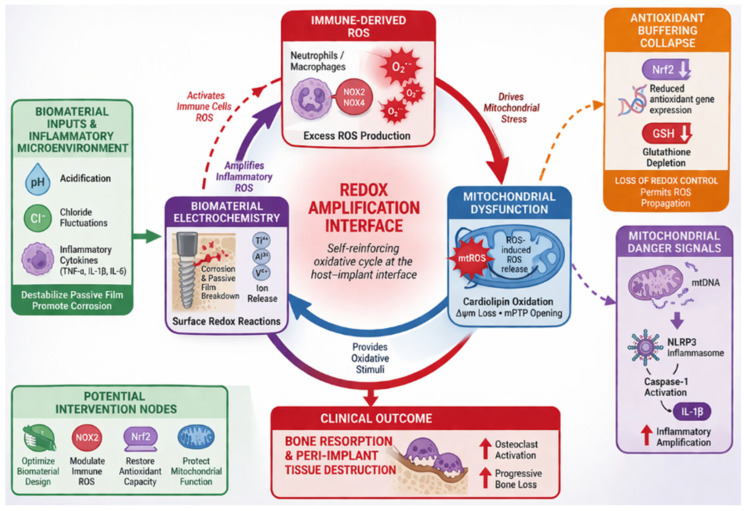
Redox amplification interface (RAI) model of peri-implant inflammation. Immune-derived reactive oxygen species (ROS), mitochondrial dysfunction, and biomaterial electrochemistry form a coupled, self-reinforcing feedback loop at the host–implant interface. Activation of NADPH oxidase generates ROS that initiate mitochondrial oxidative stress, leading to ROS-induced ROS release and amplification of mitochondrial ROS production. Concurrently, inflammatory microenvironments destabilize the implant passive oxide layer, promoting corrosion, ion release, and surface-mediated redox reactions that further increase local oxidative burden. Loss of antioxidant buffering capacity, including Nrf2 signaling impairment and glutathione depletion, removes constraints on ROS propagation and enables transition from physiological redox signaling to sustained oxidative amplification. Mitochondrial damage results in release of mitochondrial danger-associated molecular patterns, including oxidized mitochondrial DNA, which activate inflammasome signaling and further enhance inflammatory responses. These interconnected processes establish a dynamic redox amplification interface linking immune activation, mitochondrial dysfunction, and biomaterial reactivity, ultimately driving osteoclast activation, bone resorption, and peri-implant tissue destruction (Created in BioRender. Mierzejewska, A. (2016) https://BioRender.com/7fhnqv6).

From a clinical and material science perspective, this framework highlights opportunities for biomarker-guided risk stratification, targeted redox modulation, and the development of oxidation-resistant biomaterials. It supports the view of peri-implant inflammation as a redox-driven host–material interface disorder in which immune activation, mitochondrial dysfunction, and biomaterial electrochemistry form a coupled, self-reinforcing network driving sustained oxidative signaling and tissue destruction (Figure 1).

## 2. Physiological Redox Signaling in Peri-Implant Tissue Homeostasis

Physiological redox signaling constitutes the regulatory baseline of the redox amplification interface (RAI) system, integrating immune responses, mitochondrial metabolism, and skeletal remodeling under homeostatic conditions. At the biomaterial–host interface, these processes are further modulated by physicochemical properties of implant surfaces, which influence local redox dynamics and cellular responses [27,28]. Within this context, reactive oxygen species (ROS) function not as nonspecific cytotoxic byproducts, but as tightly regulated signaling mediators whose spatial and temporal distribution coordinates adaptation to microbial exposure, mechanical loading, and tissue repair [29,30].

At the molecular level, redox signaling is mediated primarily through reversible oxidative modification of redox-sensitive cysteine residues within regulatory proteins. Post-translational modifications such as cysteine sulfenylation, S-nitrosylation, and S-glutathionylation act as dynamic molecular switches controlling the activity of signaling enzymes, transcription factors, and metabolic regulators [31,32]. Through these mechanisms, ROS modulate key pathways including MAPK, NF-κB, and PI3K-Akt signaling, enabling precise regulation of immune activation, angiogenesis, cellular metabolism, and differentiation without inducing irreversible oxidative injury [29,30].

Innate immune cells represent a major source of physiological ROS at the peri-implant interface. Activation of NADPH oxidase complexes, particularly NOX2 and NOX4, generates transient superoxide bursts that contribute to antimicrobial defense while simultaneously tuning inflammatory signaling thresholds [33,34]. When antioxidant buffering capacity is preserved, these ROS signals remain spatially confined and rapidly neutralized, preventing propagation of oxidative damage to surrounding tissues [29,34]. These tightly regulated ROS bursts define the physiological threshold that, when exceeded, initiates transition toward pathological redox amplification within the RAI framework.

Mitochondria provide an additional, highly regulated source of intracellular ROS that integrates metabolic and inflammatory signaling. Low-level superoxide generation at electron transport chain complexes I and III supports mitochondrial redox signaling and coordinates cellular metabolic adaptation [35,36]. Mitochondrial ROS further influence immune cell polarization and regulate transitions between pro-inflammatory and resolution-associated macrophage phenotypes, linking bioenergetic state with immune function [36,37]. Under physiological conditions, mitochondrial ROS remain tightly controlled; however, this regulatory state represents a critical boundary beyond which mitochondria transition into dominant amplification nodes within the RAI system.

Redox signaling also plays a central role in skeletal remodeling processes essential for peri-implant stability. In osteoblast lineage cells, physiological ROS levels activate redox-sensitive transcriptional pathways including Runx2, Wnt/β-catenin, and MAP kinase signaling, promoting osteogenic differentiation and matrix mineralization [38,39]. Conversely, controlled ROS generation is required for osteoclast differentiation, where RANKL signaling induces transient oxidative signals that facilitate activation of NFATc1 and osteoclast precursor maturation [40,41]. Under physiological conditions, this balanced redox regulation ensures coupling between bone formation and resorption, maintaining structural homeostasis at the implant interface [38,40].

In addition, redox gradients regulate vascular adaptation and extracellular matrix remodeling during peri-implant healing. ROS-dependent stabilization of hypoxia-inducible factor signaling promotes angiogenesis and endothelial cell migration, while fibroblast redox signaling coordinates extracellular matrix synthesis and tissue integration [42,43].

These physiological processes are tightly constrained by integrated antioxidant defense systems that preserve cellular redox equilibrium. Enzymatic antioxidant networks—including superoxide dismutases, catalase, peroxiredoxins, and glutathione peroxidases—rapidly detoxify excess reactive species and prevent uncontrolled oxidative propagation [29,31]. Central to this regulation is the Nrf2-dependent transcriptional program, which controls expression of antioxidant enzymes, glutathione biosynthesis pathways, and metabolic systems maintaining intracellular reducing capacity [44,45].

The glutathione system constitutes a principal intracellular redox buffer that maintains thiol homeostasis and protects protein cysteine residues from irreversible oxidation [31,46]. Efficient recycling of oxidized glutathione (GSSG) to its reduced form (GSH) via NADPH-dependent glutathione reductase preserves intracellular redox potential and stabilizes redox-sensitive signaling networks. In parallel, mitochondrial quality control mechanisms—including mitophagy and mitochondrial biogenesis—ensure removal of damaged organelles and maintenance of functional mitochondrial populations capable of sustaining controlled redox signaling [47]. Maintenance of this antioxidant buffering capacity is therefore essential for constraining redox signaling within physiological limits and preventing transition into self-sustaining oxidative amplification states as defined in the RAI model.

When these regulatory systems remain intact, peri-implant tissues maintain controlled redox signaling compatible with tissue repair and integration [28,29]. However, persistent inflammatory stimulation can overwhelm antioxidant buffering capacity, destabilize mitochondrial function, and shift physiological redox signaling toward pathological oxidative amplification. Within the RAI framework, this transition represents a critical threshold event that enables coupling between immune-derived ROS, mitochondrial dysfunction, and biomaterial-driven oxidative inputs, thereby initiating the self-reinforcing amplification loops described in subsequent sections. The key distinctions between physiological redox signaling and pathological oxidative dysregulation in peri-implant tissues are summarized in Table 1.

## 3. Redox Buffering Collapse and Immune–Mitochondrial Amplification in Peri-Implant Inflammation

Within the redox amplification interface (RAI) framework, collapse of antioxidant buffering represents the critical transition that converts regulated redox signaling into a self-sustaining amplification system. This shift enables coupling between immune-derived ROS, mitochondrial dysfunction, and biomaterial-driven oxidative inputs, transforming transient inflammatory responses into persistent, self-reinforcing oxidative circuits.

### 3.1. Failure of Antioxidant Buffering: Nrf2 Exhaustion and Glutathione Depletion

Persistent inflammatory stimulation within peri-implant tissues, in combination with biomaterial-induced electrochemical stress, progressively overwhelms endogenous antioxidant systems that normally constrain physiological redox signaling [48,49,50]. Within the redox amplification interface (RAI) framework, this failure of antioxidant buffering represents a critical transition point at which regulated redox signaling loses its spatial and temporal control, enabling propagation of oxidative stress across cellular compartments.

A central regulator of cellular redox resilience is the Nrf2-Keap1 signaling pathway, which coordinates transcription of antioxidant enzymes, glutathione biosynthesis components, and detoxification networks responsible for maintaining intracellular reducing capacity [51,52,53]. Under homeostatic conditions, Nrf2 activity is tightly controlled by Keap1-mediated ubiquitination and proteasomal degradation [51]. Oxidative or electrophilic modification of critical Keap1 cysteine residues disrupts this interaction, enabling Nrf2 stabilization, nuclear translocation, and activation of cytoprotective gene programs involved in glutathione synthesis, NADPH regeneration, and oxidative stress defense [51,52,53].

However, chronic inflammatory conditions characterized by sustained ROS exposure and persistent cytokine signaling impair Nrf2 activation and limit induction in antioxidant gene expression, thereby reducing cellular redox resilience [54,55,56]. In parallel, intracellular glutathione pools—the primary thiol-based redox buffering system—become progressively depleted through continuous detoxification of hydrogen peroxide, lipid peroxides, and metal-catalyzed radical species [57,58]. Impaired NADPH-dependent regeneration of reduced glutathione (GSH) further shifts intracellular redox potential toward a more oxidized state [57,59].

This altered redox environment sensitizes mitochondrial membranes, redox-sensitive signaling proteins, and metabolic enzymes to oxidative modification, lowering the threshold for mitochondrial dysfunction [59,60]. Within the RAI framework, this loss of antioxidant buffering does not merely increase oxidative stress but functionally removes the regulatory constraints that normally prevent propagation of ROS, thereby priming the system for mitochondrial amplification and transition into self-sustaining oxidative signaling.

### 3.2. Mitochondrial Dysfunction and ROS-Induced ROS Release as the Central Amplification Engine

Following collapse of antioxidant buffering, mitochondria become both primary targets and dominant amplifiers of oxidative stress within the RAI system [61,62]. Excess cytosolic ROS damages mitochondrial DNA, oxidatively modifies electron transport chain (ETC) proteins, and disrupts structural organization of respiratory complexes, particularly complexes I and III [62,63]. These alterations impair electron transfer efficiency and increase electron leakage, resulting in elevated mitochondrial superoxide production [63,64].

Importantly, this transition represents a qualitative shift in system behavior: mitochondria no longer function as regulated signaling organelles but instead become persistent sources of oxidative output, effectively decoupling ROS generation from the initiating inflammatory stimulus. A central mechanism linking mitochondrial dysfunction to escalating oxidative stress is ROS-induced ROS release, whereby initial oxidative insults destabilize mitochondrial redox homeostasis and trigger sustained mitochondrial ROS production independent of the original trigger [65]. ROS-induced ROS release therefore constitutes the core amplification mechanism within the RAI framework, enabling exponential propagation of oxidative stress once mitochondrial instability is established.

Oxidative modification of cardiolipin, a key phospholipid of the inner mitochondrial membrane, disrupts ETC organization and promotes further electron leakage, reinforcing mitochondrial ROS generation [66,67,68]. In parallel, oxidative injury to mitochondrial membranes may induce opening of the mitochondrial permeability transition pore (mPTP), leading to loss of membrane potential, osmotic swelling, and release of mitochondrial components into the cytosol [69,70]. In addition to ROS amplification, mitochondrial outer membrane permeabilization leads to the release of cytochrome c (Cyt-C), a key initiator of the intrinsic apoptotic pathway. Under conditions of severe mitochondrial dysfunction and ATP depletion, classical apoptosis may shift toward aponecrosis or necrosis. These forms of cell death are associated with loss of membrane integrity and release of pro-inflammatory intracellular contents, further exacerbating inflammatory tissue damage within the RAI framework [19,21].

Mitochondrial dynamics are also profoundly altered under oxidative stress. Increased DRP1-dependent fission and impaired OPA1-mediated fusion result in mitochondrial fragmentation and accumulation of structurally compromised organelles [71,72,73]. Concurrently, impaired mitophagy and reduced mitochondrial biogenesis limit removal and replacement of damaged mitochondria, leading to persistence of dysfunctional mitochondrial populations [74,75].

**Figure 2:**
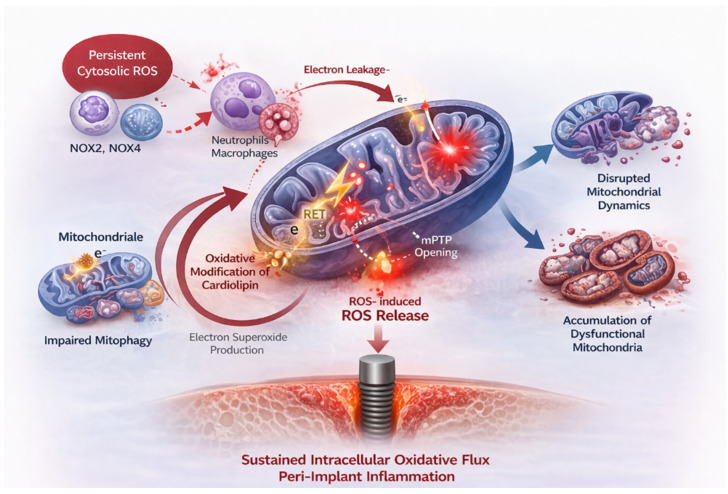
Mitochondrial oxidative amplification as a self-sustaining driver of peri-implant inflammation. Persistent cytosolic ROS damages electron transport chain complexes, increasing electron leakage and mitochondrial superoxide production. Oxidative modification of cardiolipin and mitochondrial membrane depolarization promote ROS-induced ROS release and opening of the mitochondrial permeability transition pore. Concurrent disruption of mitochondrial dynamics and impaired mitophagy lead to accumulation of dysfunctional mitochondria that sustain intracellular oxidative flux independently of the initiating inflammatory stimulus (Created in BioRender. Mierzejewska, A. (2016) https://BioRender.com/7fhnqv6).

Once established, this state of mitochondrial dysfunction acts as a self-sustaining oxidative amplification engine that converts transient inflammatory stimuli into continuous intracellular ROS flux. Within the RAI framework, mitochondria therefore function as the central amplification node integrating immune-derived oxidative signals with persistent redox dysregulation. This transition effectively shifts the system from regulated signaling to a pathological steady state characterized by sustained oxidative output and progressive cellular damage. As illustrated in Figure 2, these processes collectively transform mitochondria from physiological signaling organelles into dominant drivers of chronic inflammatory activation.

This transition positions mitochondria as self-sustaining sources of oxidative flux, effectively decoupling inflammatory persistence from the initiating stimulus.

### 3.3. Redox-Driven Immune–Mitochondrial Inflammatory Feedback Loop

Mitochondrial oxidative amplification directly interfaces with innate immune signaling networks to establish a self-reinforcing inflammatory feedback loop within the RAI system [76,77]. Microbial biofilms at the implant surface activate neutrophils and macrophages via pattern recognition receptor signaling [78], leading to NADPH oxidase activation and generation of superoxide as part of the antimicrobial oxidative burst [79,80].

Under conditions of preserved antioxidant buffering, these ROS signals remain transient and spatially confined [81]. However, once mitochondrial vulnerability develops, NOX-derived ROS act as initiating triggers for mitochondrial amplification, dramatically increasing intracellular ROS flux [76,82]. In turn, mitochondrial ROS activate redox-sensitive transcription factors such as NF-κB and AP-1, promoting expression of pro-inflammatory cytokines including TNF-α, IL-1β, and IL-6 [83,84].

These cytokines further enhance NADPH oxidase activity and inflammatory cell recruitment, reinforcing ROS generation and sustaining the amplification cycle [80,83]. Simultaneously, oxidative mitochondrial injury leads to release of mitochondrial danger-associated molecular patterns, including oxidized mitochondrial DNA, which activate the NLRP3 inflammasome, resulting in caspase-1 activation and maturation of IL-1β [85,86,87]. This process further intensifies inflammatory signaling and amplifies osteoclastogenic pathways.

Within bone remodeling compartments, elevated ROS amplify RANKL-dependent signaling pathways that drive osteoclast differentiation via NFATc1 activation, while concurrently suppressing osteoblast survival and osteogenic transcriptional programs [88,89,90,91]. This redox-mediated uncoupling of bone remodeling shifts the balance toward resorptive dominance and progressive peri-implant bone loss.

Collectively, these processes establish a closed-loop amplification system in which immune-derived ROS trigger mitochondrial dysfunction, mitochondrial ROS amplify inflammatory signaling, and cytokine-driven NADPH oxidase activation further reinforces ROS production. Within the RAI framework, this feedback loop represents a self-sustaining pathogenic circuit that progressively reduces dependence on microbial stimuli and contributes to chronicity and therapeutic resistance.

Through these interconnected mechanisms, collapse of antioxidant buffering initiates mitochondrial amplification that integrates with immune signaling to generate a self-sustaining immune–mitochondrial inflammatory circuit. Within the RAI framework, this closed-loop system functions as a central pathogenic engine that progressively reduces dependence on microbial stimuli and contributes to chronicity and therapeutic resistance [76,83,85].

The multi-layered mechanisms underlying redox buffering collapse and subsequent amplification within the RAI system are summarized in Table 2.

## 4. Biomaterial-Driven Redox Amplification at the Host–Implant Interface

Metallic biomaterials at the implant interface function as active electrochemical modulators of the local microenvironment, directly contributing to redox amplification rather than merely serving as passive structural supports [92,93]. Within the redox amplification interface framework, the implant surface becomes an integral component of a coupled biological–electrochemical system in which immune-derived oxidants, local chemical conditions, and material properties dynamically interact to shape oxidative stress propagation [94,95]. Under inflammatory conditions, microenvironments factors such as acidic pH, elevated chloride concentrations, and increased flux of reactive oxygen and nitrogen species pH, elevated chloride concentrations, and increased flux of reactive oxygen and nitrogen species may destabilize protective oxide layers on metallic implants, promoting corrosion, ion release, and surface-mediated redox reactions [96,97,98].

Corrosion processes at the implant interface result in the release of titanium ions and nanoscale particulate debris into surrounding tissues [99,100,101]. Within the RAI framework, these material-derived species act as secondary amplifiers of oxidative stress rather than inert byproducts [102]. Transition metal ions, surface defects, and nanoscale particles can catalyze redox cycling reactions, promoting generation of reactive oxygen species and propagation of radical-mediated oxidative processes [103,104,105,106]. Although titanium exhibits relatively low intrinsic redox activity, nanoscale particles, alloying elements, and defect-rich surfaces may significantly enhance localized catalytic activity under inflammatory conditions [107,108,109].

At the cellular level, internalization of titanium particles by macrophages and neutrophils triggers NADPH oxidase activation and enhances mitochondrial ROS production, thereby reinforcing immune–mitochondrial amplification pathways [110,111,112,113]. In parallel, metal particles and ions may activate inflammasome signaling, further increasing production of pro-inflammatory cytokines and sustaining inflammatory recruitment [114,115,116,117]. Persistent exposure to corrosion-derived debris can therefore maintain inflammatory signaling even in the absence of substantial microbial stimulation [118,119].

Beyond particle-mediated effects, the implant surface itself functions as a catalytic redox interface. Interactions between immune-derived oxidants and metallic surfaces facilitate electron transfer processes that promote radical propagation and oxidative modification of surrounding biomolecules [120,121]. Metal ions released during corrosion may further participate in Fenton-like reactions, generating highly reactive hydroxyl radicals that amplify lipid peroxidation and protein oxidation within peri-implant tissues [122,123].

Importantly, the extent to which biomaterials contribute to redox amplification is modulated by their intrinsic electrochemical stability. Materials with higher corrosion resistance, such as titanium alloys incorporating niobium, tantalum, or zirconium, exhibit enhanced passive layer stability and reduced ion release under inflammatory conditions [94,95]. In contrast, electrochemically inert ceramics such as zirconia display minimal redox reactivity across a wide range of conditions, limiting their contribution to oxidative amplification processes [25,107].

Within the RAI framework, biomaterials can therefore be positioned along a functional spectrum of redox reactivity, where material composition, surface properties, and electrochemical stability determine their capacity to participate in oxidative signaling networks. Crucially, the biological impact of these material-derived oxidative inputs depends on host redox resilience. In individuals with preserved antioxidant buffering capacity, corrosion-driven oxidative stimuli may be effectively neutralized. In contrast, in hosts with impaired Nrf2 signaling, glutathione depletion, or increased mitochondrial susceptibility, even modest electrochemical perturbations may trigger self-reinforcing immune–mitochondrial amplification loops [29,59].

**Figure 3:**
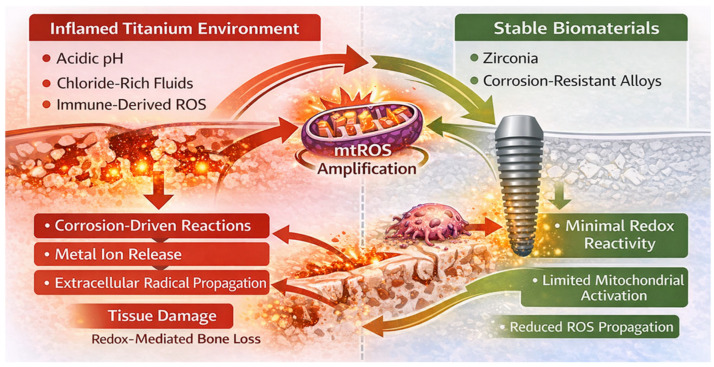
Biomaterial electrochemical reactivity as a modulator of redox amplification within the redox amplification interface (RAI) system. Inflammatory microenvironments destabilize titanium passive oxide layers, promoting corrosion-driven redox reactions, ion release, and extracellular radical propagation. These material-derived oxidative inputs interact with immune-derived ROS and mitochondrial dysfunction to reinforce intracellular amplification pathways. Within the RAI framework, biomaterials function as active modulators of oxidative stress, contributing to a coupled feedback system linking electrochemical processes with immune–mitochondrial signaling and sustained inflammatory activation (Created in BioRender. Mierzejewska, A. (2016) https://BioRender.com/7fhnqv6).

Collectively, these observations position biomaterials as active components of a redox-amplified host–material interface system in which electrochemical behavior directly modulates immune activation, mitochondrial function, and inflammatory persistence [25,59]. As illustrated in Figure 3, biomaterial electrochemical reactivity integrates with immune-derived ROS and mitochondrial dysfunction to form a coupled redox system in which corrosion-driven oxidative inputs reinforce intracellular amplification pathways, thereby sustaining inflammatory signaling within the RAI framework.

### 4.1. Corrosion Electrochemistry and Oxidative Microenvironments

Within the redox amplification interface model, corrosion electrochemistry represents a primary material-driven source of oxidative amplification at the implant surface. Under inflammatory conditions, destabilization of the titanium passive oxide layer promotes localized corrosion reactions that directly contribute to the formation of redox-active microenvironments [101,102].

Acidic pH, elevated chloride concentrations, and high flux of immune-derived oxidants shift the electrochemical equilibrium at the implant surface, facilitating breakdown of the passive TiO_2_ layer and enhancing anodic dissolution of titanium [102,103,104]. These processes are associated with the generation of corrosion currents, alterations in corrosion potential, and the release of soluble titanium ions as well as nanoscale particulate debris into surrounding tissues [103,105]. In this context, implant corrosion should be considered not merely as a secondary degradation phenomenon but as an active contributor to oxidative microenvironment formation [101,104].

Importantly, electrochemical reactions occurring at metal surfaces may directly catalyze redox processes leading to ROS generation [106,107]. Metal ions released during corrosion can participate in redox cycling reactions, promoting radical formation and propagation of oxidative chemistry within the peri-implant microenvironment [106,108]. Although titanium exhibits relatively low intrinsic redox activity, the presence of surface defects, alloying elements, and nanoscale particulate debris can enhance localized catalytic activity, particularly under inflammatory conditions [105,109].

Through these mechanisms, electrochemical degradation of implant surfaces transforms the implant–tissue interface into a dynamically regulated redox-active microenvironment. Within the RAI framework, this material-driven oxidative input acts in concert with immune-derived ROS and mitochondrial dysfunction to amplify oxidative stress and sustain inflammatory signaling in peri-implant tissues [101,106,109].

### 4.2. Metal Particle Immunology and Inflammatory ROS Generation

Corrosion-derived metal ions and nanoscale titanium particles are readily internalized by macrophages, neutrophils, and fibroblasts present within inflamed peri-implant tissues [110]. Cellular uptake of these particles activates innate immune signaling pathways and promotes reactive oxygen species production through multiple mechanisms [111,112].

Phagocytosis of titanium particles stimulates NADPH oxidase activation in macrophages, leading to enhanced superoxide generation [113]. In parallel, intracellular metal particles disrupt mitochondrial membrane potential and stimulate mitochondrial ROS production, thereby reinforcing mitochondrial oxidative amplification pathways described in earlier sections. Metal particles may also activate inflammasome signaling pathways in macrophages, contributing to maturation of pro-inflammatory cytokines such as IL-1β and further intensifying inflammatory recruitment [114,115,116,117].

Importantly, persistent exposure to particulate debris can sustain chronic immune activation even in the absence of substantial microbial stimulation [118]. Within the RAI framework, this particle-driven immune activation represents a key amplification layer that links biomaterial degradation to mitochondrial dysfunction and self-sustaining inflammatory circuits [119].

### 4.3. Implant Surface Redox Catalysis and Interfacial Oxidative Processes

In addition to particle-mediated immune activation, the implant surface itself functions as a redox-active catalytic interface within the RAI framework. Reactive oxygen species generated by inflammatory cells can interact with metallic implant surfaces, where electrochemical reactions facilitate electron transfer processes, radical propagation, and oxidative modification of surrounding biomolecules [50,121].

Metal ions released during corrosion can catalyze redox reactions that promote formation of highly reactive radical species capable of initiating lipid peroxidation and oxidative modification of extracellular matrix components [58,123]. These ions may further contribute to oxidative amplification through Fenton-like reactions generating highly reactive hydroxyl radicals [49].

Surface microtopography plays a critical role in modulating these processes. Roughened titanium surfaces, widely used to enhance osseointegration, increase the electrochemically active surface area and create confined microdomains in which acidic metabolites and reactive species accumulate [23,92]. Under inflammatory conditions, these microenvironments facilitate localized corrosion reactions and amplify surface-driven oxidative chemistry [25,101].

Conversely, biomaterials with greater electrochemical stability attenuate these redox interactions. Ceramic materials such as zirconia exhibit minimal electrochemical reactivity across a wide range of pH and oxidative conditions, limiting corrosion-driven ion release and catalytic redox activity [25,107]. Similarly, advanced titanium alloys incorporating elements such as niobium, tantalum, or zirconium demonstrate improved passive layer stability and reduced corrosion susceptibility [94,95].

### 4.4. Integration of Material Reactivity with Host Redox Susceptibility

The biological impact of implant-derived oxidative stimuli is critically modulated by host redox resilience, representing a key coupling node within the RAI model [29,59]. In individuals with preserved antioxidant buffering capacity and efficient mitochondrial quality control, moderate corrosion-derived oxidative inputs may be neutralized without substantial pathological consequences [29]. In contrast, in hosts exhibiting compromised antioxidant systems, impaired Nrf2 signaling, or heightened mitochondrial susceptibility, even modest material-derived oxidative stimuli may trigger self-reinforcing immune–mitochondrial amplification loops [45,50].

This interaction indicates that implant materials should not be considered biologically inert structural components, but rather dynamic participants in a redox-active interface whose behavior depends on both material electrochemistry and host oxidative phenotype [27,29]. Within this context, corrosion-driven redox reactions, particle-mediated immune activation, and mitochondrial oxidative amplification form an integrated system linking biomaterial properties to inflammatory tissue destruction.

Collectively, these observations position implant materials along a functional spectrum of redox reactivity, where electrochemical stability becomes a critical determinant of inflammatory amplification and disease progression in peri-implant tissues [25,95]. Together, corrosion electrochemistry, particle-driven immune activation, and surface-mediated redox catalysis constitute a material-dependent layer of the RAI model, directly coupling implant properties with mitochondrial dysfunction and sustained inflammatory signaling.

## 5. Clinical Redox Phenotypes and Oxidative Biomarkers as Functional Readouts

Within the redox amplification interface (RAI) framework, oxidative biomarkers represent functional readouts of distinct mechanistic layers linking immune activation, mitochondrial dysfunction, and biomaterial-driven redox perturbations. Rather than serving as nonspecific indicators of inflammation, these biomarkers can be interpreted as system-level signatures reflecting specific stages of redox amplification, ranging from early antioxidant buffering failure to sustained immune–mitochondrial feedback loops. The framework outlined above predicts that these coupled processes give rise to clinically distinct redox phenotypes that can be captured through integrated oxidative biomarker profiling [5,12]. In this context, oxidative biomarkers should not be interpreted as nonspecific indicators of inflammation, but rather as mechanistically informative signatures reflecting specific stages of redox amplification, ranging from early antioxidant buffering failure to sustained immune–mitochondrial oxidative loops [13,35].

### 5.1. Oxidative Damage Markers Reflecting Mitochondrial and Inflammatory ROS Activity

Biomarkers of oxidative macromolecular damage provide integrative measures of cumulative reactive oxygen and nitrogen species exposure within peri-implant tissues. Within the RAI framework, these markers primarily reflect downstream consequences of mitochondrial amplification and sustained immune-derived ROS activity.

Oxidative DNA damage products such as 8-hydroxy-2′-deoxyguanosine (8-OHdG) reflect prolonged mitochondrial and nuclear ROS exposure and are frequently elevated in conditions characterized by persistent oxidative stress [5,12,13]. Due to limited repair capacity and lack of protective histones, mitochondrial DNA is particularly susceptible to oxidative damage; thus, elevated 8-OHdG may serve as an indirect indicator of mitochondrial dysfunction and ROS-induced ROS release mechanisms [19,21].

Lipid peroxidation products, including malondialdehyde (MDA) and 4-hydroxynonenal (4-HNE), reflect propagation of radical-mediated damage within cellular and mitochondrial membranes [58]. These reactive aldehydes form covalent adducts with proteins and nucleic acids, amplifying cellular dysfunction and reinforcing inflammatory signaling [60,68].

Protein oxidative modifications provide complementary insight into sustained redox imbalance. Protein carbonylation represents a relatively stable marker of oxidative protein damage, while nitrotyrosine formation reflects peroxynitrite-mediated nitrative stress arising from dysregulated interactions between nitric oxide and superoxide [13,35,98]. Together, these biomarkers capture cumulative oxidative injury across DNA, lipids, and proteins within inflamed peri-implant tissues. Within the RAI framework, these damage-associated biomarkers primarily reflect downstream consequences of mitochondrial amplification and sustained immune-derived ROS activity, thereby serving as indicators of established oxidative propagation rather than early initiating events.

### 5.2. Biomarkers of Redox Buffering Capacity and Antioxidant Depletion

While oxidative damage markers reflect downstream consequences of ROS excess, biomarkers of antioxidant systems provide insight into upstream determinants of redox resilience within the RAI system.

Measurement of total antioxidant capacity offers a global estimate of systemic and local reserves available to neutralize reactive species [12,29]. More mechanistically informative indicators include the glutathione redox couple. The ratio between reduced and oxidized glutathione (GSH/GSSG) represents a sensitive indicator of intracellular redox potential and thiol buffering capacity [29,55]. Declining GSH/GSSG ratios reflect exhaustion of antioxidant defenses and indicate a shift toward an oxidized intracellular environment that facilitates mitochondrial vulnerability and oxidative amplification [17,59]. Alterations in enzymatic antioxidant systems—including superoxide dismutases, catalase, and glutathione peroxidases—further reflect adaptive or exhausted responses to sustained oxidative pressure [12,54]. Integration of these measurements with oxidative damage markers enables mapping of both upstream buffering failure and downstream oxidative injury within a unified redox framework. These biomarkers therefore provide insight into upstream regulatory failure within the RAI system, identifying loss of redox buffering capacity as a critical transition point preceding mitochondrial amplification.

### 5.3. Emerging Approaches: Redox Proteomics and Oxidative Signaling Signatures

Advances in redox biology highlight the importance of reversible oxidative modifications of protein cysteine residues in regulating cellular signaling networks. Redox proteomics enables detection of post-translational modifications such as S-nitrosylation, S-glutathionylation, and sulfenylation across complex protein networks [30,31]. Although these approaches have not yet been widely applied in peri-implant disease, they offer significant potential for identifying signaling nodes involved in redox amplification. Within the RAI framework, redox proteomic profiling may enable direct mapping of oxidative modifications affecting immune signaling pathways, mitochondrial proteins, and bone remodeling regulators, thereby providing mechanistic resolution beyond conventional biomarker panels [13,35]. Redox proteomic signatures may enable direct mapping of signaling nodes affected by oxidative modifications, providing mechanistic resolution of amplification pathways beyond conventional biomarker approaches.

### 5.4. Local Versus Systemic Oxidative Biomarkers

Interpretation of oxidative biomarkers requires consideration of the biological compartment in which measurements are obtained. Local biomarkers assessed in peri-implant crevicular fluid or peri-implant tissues more directly reflect redox processes occurring within the inflammatory microenvironment at the implant interface [5,12]. In contrast, systemic biomarkers measured in plasma or saliva capture broader oxidative states influenced by systemic inflammation, metabolic status, and environmental factors [11,12].

Integration of local and systemic measurements provides complementary insight into the RAI system. Local biomarkers reflect active interface-level amplification processes, whereas systemic markers may indicate host-level redox susceptibility that predisposes individuals to exaggerated inflammatory responses [5,12]. This distinction aligns with the RAI model, in which local biomarkers reflect interface-level amplification processes, while systemic markers indicate host-level redox susceptibility that modulates overall system behavior.

### 5.5. Redox Phenotypes and Translational Implications

Integration of oxidative damage markers, antioxidant capacity measurements, and emerging redox proteomic signatures supports the existence of distinct clinical redox phenotypes within peri-implant disease. Low-risk phenotypes are characterized by preserved antioxidant buffering and limited oxidative propagation, allowing inflammatory responses to remain self-limited despite microbial challenge [5,12]. Intermediate phenotypes exhibit early depletion of antioxidant reserves accompanied by moderate increases in oxidative damage markers, indicating vulnerability to further amplification [29,59]. In contrast, high-risk oxidative phenotypes display pronounced oxidative DNA damage, lipid peroxidation, protein nitration, and depletion of antioxidant systems, consistent with sustained immune–mitochondrial amplification loops as defined in the RAI model [45,50].

Recognition of these phenotypes has direct translational implications. Biomarker-guided redox stratification may enable differentiation between infection-dominant inflammation and disease driven primarily by oxidative amplification mechanisms [5,12]. In clinical scenarios where oxidative burden disproportionately exceeds microbial indices, therapeutic strategies targeting redox dysregulation represent a rational adjunct to conventional antimicrobial approaches [29,54].

Prospective validation of integrated biomarker panels will be required to determine their predictive value for disease progression and therapeutic response. Nevertheless, the mechanistic coherence between mitochondrial dysfunction, antioxidant buffering collapse, and clinically measurable oxidative signatures supports the feasibility of biomarker-guided redox phenotyping as a precision medicine approach in peri-implant disease. These phenotype-specific signatures provide a basis for biomarker-guided stratification of patients according to dominant mechanistic drivers within the RAI system, enabling alignment of therapeutic strategies with underlying redox dynamics rather than microbial burden alone. The principal oxidative biomarkers reflecting distinct stages of redox dysregulation are summarized in Table 3.

Recognition of redox dysregulation as a central amplification mechanism within the RAI model identifies multiple therapeutic nodes capable of interrupting the self-reinforcing immune–mitochondrial-biomaterial cycle. Rather than targeting microbial burden alone, redox-oriented strategies aim to restore physiological redox homeostasis, stabilize mitochondrial function, and limit material-driven oxidative inputs. From a translational perspective, these intervention points include modulation of ROS production, restoration of antioxidant buffering systems, protection of mitochondrial integrity, and optimization of biomaterial electrochemical stability.

## 6. Redox-Targeted Therapeutic Strategies at the Host–Implant Interface

Within the redox amplification interface (RAI) model, therapeutic interventions can be conceptualized as targeted disruptions of specific nodes within the self-reinforcing immune–mitochondrial–biomaterial system. Rather than focusing solely on microbial reduction, redox-oriented strategies aim to restore regulatory control over oxidative signaling, stabilize mitochondrial function, and limit material-driven amplification processes.

### 6.1. Modulation of Immune-Derived ROS Generation at the Biomaterial Interface

Excessive ROS generation by activated innate immune cells represents an early driver of redox amplification. NADPH oxidase complexes, particularly NOX2, play a central role in superoxide generation during inflammatory responses [33,34]. Controlled modulation of NADPH oxidase activity therefore represents a potential strategy to reduce excessive ROS production while preserving physiological antimicrobial defense [76].

Selective attenuation of excessive NOX activation may limit downstream mitochondrial oxidative amplification and inflammatory signaling. Importantly, complete suppression of ROS generation is not desirable, as physiological ROS are essential for host defense and immune regulation. Within the RAI framework, therapeutic strategies should therefore aim to recalibrate immune-derived ROS toward physiological signaling ranges rather than eliminate them entirely [34,50,76]. Pharmacological modulation of NADPH oxidase activity includes compounds such as apocynin and diphenyleneiodonium (DPI), as well as emerging isoform-selective NOX inhibitors (e.g., GSK2795039). These agents have been shown to reduce excessive ROS generation and may attenuate downstream mitochondrial oxidative amplification [33,34].

### 6.2. Restoration of Endogenous Antioxidant Capacity and Redox Buffering

A second major therapeutic axis involves restoration of endogenous antioxidant systems that define cellular redox resilience. Central to this approach is activation of the Nrf2 signaling pathway, which regulates transcription of antioxidant enzymes, glutathione biosynthesis pathways, and metabolic systems maintaining intracellular reducing capacity [17,51,54].

Pharmacologic Nrf2 activators, including dimethyl fumarate and related electrophilic compounds, promote dissociation of Nrf2 from Keap1 and induce expression of cytoprotective genes such as heme oxygenase-1 (HO-1), NAD(P)H quinone oxidoreductase 1 (NQO1), and enzymes involved in glutathione synthesis [16,56]. Enhancement of Nrf2-dependent responses may restore buffering capacity, limit oxidative propagation, and increase tissue resilience to inflammatory stress [53,54].

Complementary strategies targeting the glutathione system, either by increasing glutathione availability or enhancing NADPH-dependent recycling, may further stabilize intracellular redox potential and prevent transition toward mitochondrial dysfunction and amplification states [29,55]. Additional strategies to restore antioxidant capacity include supplementation with N-acetylcysteine (NAC), which replenishes intracellular glutathione pools, as well as activation of Nrf2 signaling by compounds such as sulforaphane. These approaches may enhance cellular redox resilience and counteract oxidative amplification at the implant–tissue interface [16,17,29].

### 6.3. Targeting Mitochondrial Oxidative Amplification and Bioenergetic Dysfunction

Given the central role of mitochondria in sustaining ROS-induced ROS release, therapeutic strategies aimed at preserving mitochondrial integrity represent an important intervention point within the redox amplification cascade [19,50]. Mitochondria-targeted antioxidants have emerged as promising candidates capable of selectively reducing mitochondrial oxidative stress while sparing physiological cytosolic redox signaling [64].

One such compound, MitoQ, consists of a ubiquinone moiety linked to a lipophilic triphenylphosphonium cation that facilitates selective accumulation within the mitochondrial matrix driven by the mitochondrial membrane potential. Once localized within mitochondria, MitoQ acts as a redox-active antioxidant capable of limiting mitochondrial lipid peroxidation, stabilizing respiratory chain function, and reducing mitochondrial ROS production [64].

Another mitochondria-directed therapeutic approach involves mitochondrial membrane-stabilizing peptides such as Elamipretide. Elamipretide selectively binds cardiolipin within the inner mitochondrial membrane, stabilizing mitochondrial cristae structure and improving electron transport chain efficiency. By preserving mitochondrial membrane integrity and limiting cardiolipin oxidation, such compounds may reduce ROS-induced ROS release and improve mitochondrial bioenergetic function under conditions of oxidative stress [66,75].

**Figure 4:**
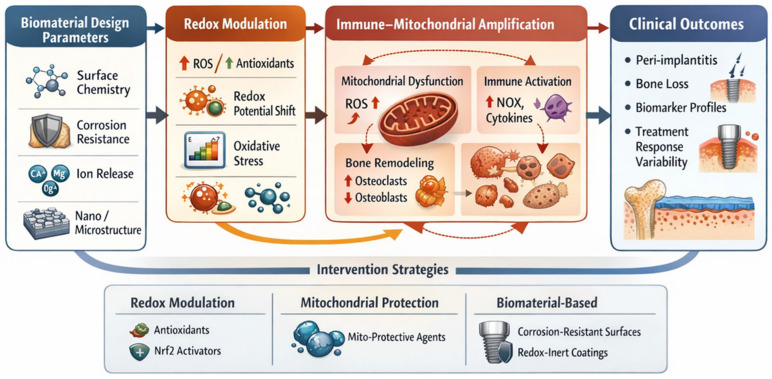
Translational framework linking biomaterial design to redox modulation and clinical outcomes at the host–implant interface. This schematic illustrates how biomaterial properties shape local redox microenvironments at the host–implant interface, thereby modulating immune activation, mitochondrial function, and bone remodeling. Electrochemical instability and corrosion processes amplify oxidative stress, promoting mitochondrial dysfunction and inflammatory signaling. These processes form a self-reinforcing feedback loop that drives peri-implant tissue destruction. Importantly, this framework highlights multiple intervention points, including biomaterial design optimization, redox modulation, and mitochondrial protection, providing a translational basis for next-generation implant strategies (Created in BioRender. Mierzejewska, A. (2016) https://BioRender.com/7fhnqv6).

In addition to direct antioxidant approaches, enhancement of mitochondrial quality control represents a complementary strategy. Promotion of mitophagy and mitochondrial biogenesis facilitates removal of damaged organelles and restoration of functional mitochondrial networks, reducing persistence of oxidative amplification [46,72]. Together, these approaches target the central amplification node of the RAI system. This positioning of mitochondria as a central amplification node aligns with the framework depicted in Figure 4, where biomaterial-induced redox imbalance converges on mitochondrial dysfunction to drive inflammatory and osteolytic responses.

### 6.4. Biomaterial Strategies to Limit Redox Amplification

In parallel with host-directed interventions, modification of implant materials represents a critical strategy to reduce material-driven oxidative inputs within the RAI system. As discussed in Section 5, electrochemical instability of metallic implant surfaces contributes to corrosion-driven redox reactions and release of pro-inflammatory metal particles.

Selection of biomaterials with enhanced electrochemical stability may attenuate these processes. Ceramic materials such as zirconia exhibit minimal redox reactivity across a wide range of conditions, limiting corrosion-driven oxidative inputs [25,107]. Advanced titanium alloys incorporating elements such as niobium, tantalum, or zirconium provide improved passive layer stability and reduced ion release [94,95].

Surface engineering strategies that minimize electrochemical heterogeneity while preserving osseointegration may further reduce localized oxidative amplification [23,25]. Within this framework, biomaterial design should be considered an active therapeutic strategy, where modulation of electrochemical properties directly influences biological outcomes.

### 6.5. Toward Biomarker-Guided Redox Therapy

Redox-targeted therapies are likely to achieve maximal efficacy when aligned with individual oxidative susceptibility profiles. Within the RAI model, oxidative biomarkers provide a basis for stratifying patients according to dominant mechanistic drivers of disease.

Patients with preserved antioxidant buffering may respond adequately to conventional antimicrobial and mechanical interventions [5,12]. In contrast, individuals exhibiting high-risk redox phenotypes—characterized by antioxidant depletion, mitochondrial dysfunction, and sustained oxidative damage—may benefit from adjunctive therapies targeting redox imbalance and mitochondrial integrity [29,41,59].

Integration of oxidative biomarker profiling with targeted interventions therefore enables a precision medicine approach in which therapeutic strategies are tailored to both microbial burden and host redox phenotype. This framework positions redox modulation, mitochondrial protection, and biomaterial optimization as complementary components of next-generation peri-implant disease management.

## 7. Testable Predictions of the Redox Amplification Interface Model

The severity of peri-implant inflammation may correlate with markers of mitochondrial oxidative damage and amplification. This relationship can be evaluated by assessing mitochondrial ROS production, mitochondrial membrane potential, and oxidative DNA damage markers such as 8-OHdG in peri-implant tissues. Increased mitochondrial dysfunction would be expected to be associated with enhanced activation of redox-sensitive inflammatory pathways and inflammasome signaling.

Biomaterial corrosion dynamics are likely to modulate local oxidative stress intensity. This can be investigated by correlating electrochemical parameters and ion release profiles with oxidative biomarkers, including lipid peroxidation products and protein nitration markers. Within the RAI framework, increased corrosion activity may be associated with elevated oxidative stress and mitochondrial dysfunction.

Modulation of antioxidant buffering capacity represents another key regulatory axis influencing both intracellular and material-driven amplification processes. Pharmacological activation of Nrf2 signaling or restoration of glutathione levels may reduce mitochondrial ROS production and attenuate inflammatory signaling. These effects may be evaluated using antioxidant interventions and functional readouts of mitochondrial and inflammatory activity.

Biomaterials characterized by enhanced electrochemical stability may exhibit a reduced capacity to sustain redox amplification loops. Comparative analyses of different implant materials could reveal lower mitochondrial ROS production, reduced oxidative biomarker levels, and attenuated inflammatory responses under comparable conditions.

Integration of oxidative biomarkers with material-specific parameters may enable stratification of patients into distinct redox phenotypes with predictive value for disease progression and therapeutic response. Longitudinal studies combining biomarker profiling with clinical outcomes and implant material characteristics would provide a robust validation of this systems-level framework.

Collectively, these considerations define a multi-scale experimental framework linking mitochondrial function, and biomaterial electrochemistry in peri-implant inflammation.

## 8. Experimental Validation Strategies for the RAI Framework

Mitochondrial amplification can be assessed through measurement of mitochondrial ROS production using probes such as MitoSOX, combined with evaluation of electron transport chain function and bioenergetic profiling (e.g., Seahorse assays). Detection of cardiolipin oxidation and mitochondrial membrane potential changes may further define the transition toward ROS-induced ROS release.

Redox buffering capacity can be quantified through analysis of glutathione redox status (GSH/GSSG ratio), NADPH availability, and expression of Nrf2-regulated antioxidant genes. These parameters provide insight into the threshold at which physiological redox signaling shifts toward sustained oxidative amplification.

Inflammatory coupling may be evaluated by assessing activation of redox-sensitive signaling pathways, including NF-κB and NLRP3 inflammasome activation, in relation to mitochondrial ROS levels and oxidative damage markers such as 8-OHdG and lipid peroxidation products.

Biomaterial-driven oxidative inputs can be characterized using electrochemical techniques, including electrochemical impedance spectroscopy and corrosion potential measurements, coupled with quantification of ion release via inductively coupled plasma mass spectrometry (ICP-MS). Correlating these parameters with local oxidative biomarkers would enable direct assessment of material–redox coupling.

Integration of these measurements within in vitro or ex vivo models of the implant interface would allow testing of whether mitochondrial dysfunction, antioxidant depletion, and corrosion-derived oxidative inputs act synergistically to sustain inflammatory signaling, as predicted by the RAI framework.

## 9. Discussion

The framework presented in this review redefines peri-implant inflammatory disease as a redox-amplified host–material interface pathology, in which biomaterial electrochemistry is mechanistically coupled to immune activation and mitochondrial dysfunction within a unified redox amplification interface (RAI) system [14,19,23,24]. Peri-implant tissue breakdown can be understood as the emergent outcome of dysregulated redox networks in which immune-derived ROS, mitochondrial amplification, and material-driven oxidative inputs converge to generate self-sustaining inflammatory signaling [45,48,50].

This redox-centered perspective provides a mechanistic explanation for the well-recognized clinical heterogeneity observed in peri-implant disease. Although microbial biofilms initiate inflammatory responses, comparable microbial burden frequently results in divergent clinical trajectories ranging from stable peri-implant conditions to rapid bone loss [2,3,10]. Within the RAI framework, this variability reflects differences in host redox resilience, mitochondrial susceptibility to oxidative injury, and the capacity of antioxidant systems to constrain amplification of ROS signaling [29,59].

Mitochondria function as central amplification nodes within the RAI system, where loss of antioxidant buffering promotes sustained oxidative stress and activation of redox-sensitive inflammatory pathways [20,45,50]. Through cardiolipin oxidation, mitochondrial permeability transition, and release of mitochondrial danger-associated molecular patterns such as oxidized mitochondrial DNA, mitochondria become both drivers and targets of inflammatory amplification [21,66,70]. Once established, these mitochondrial circuits can sustain inflammatory signaling independently of the initiating microbial stimulus, contributing to chronicity and therapeutic resistance [43,83,87].

Importantly, peri-implant tissues represent a unique biological context in which host inflammatory processes directly interact with implanted biomaterials. Electrochemical instability of metallic implant surfaces under inflammatory conditions introduces an additional layer of redox complexity that is largely absent from most chronic inflammatory diseases [23,26,97]. Corrosion-driven ion release, particulate debris, and surface-catalyzed redox reactions provide continuous oxidative inputs that reinforce immune–mitochondrial amplification loops [25,107,123]. Within the RAI model, the implant–tissue interface therefore functions as a dynamically regulated redox-active system rather than a passive structural environment [27].

Chronic activation of the foreign body response at the implant interface may also lead to excessive fibrosis, which is frequently associated with implant complications and failure. Fibrotic encapsulation reflects persistent dysregulation of tissue remodeling processes and may be influenced by oxidative stress-dependent signaling pathways that regulate fibroblast activation and extracellular matrix deposition. In this context, redox amplification may contribute not only to inflammatory persistence but also to maladaptive fibrotic responses at the host–implant interface [124].

Integration of biomaterial electrochemistry with immune and mitochondrial redox biology highlights the need for a paradigm shift in the conceptualization of peri-implant disease. While microbial dysbiosis remains an essential initiating factor, the persistence, severity, and therapeutic refractoriness of disease appear to be governed by host oxidative susceptibility and amplification dynamics rather than microbial burden alone [48,49].

The translational implications of this framework are substantial. From a translational perspective, these findings support the development of biomaterials specifically engineered to limit redox amplification at the host–implant interface. First, recognition of distinct oxidative phenotypes supports biological stratification of peri-implant disease. Biomarker panels reflecting oxidative damage, antioxidant depletion, and mitochondrial dysfunction may enable identification of patients at risk of redox-driven amplification [5,12,59]. Second, identification of mitochondria as a central amplification node highlights mitochondrial protection and redox modulation as rational therapeutic strategies [46,64,66]. Third, biomaterial design emerges as a critical and underappreciated therapeutic dimension, where optimization of electrochemical stability may directly reduce oxidative inputs at the implant interface [25,94].

Several limitations of the current evidence base must be acknowledged. Direct causal evidence linking oxidative dysregulation to peri-implant tissue destruction in humans remains limited, with most available studies being cross-sectional in nature [11,12]. Furthermore, aspects of the proposed framework rely on extrapolation from other chronic inflammatory and metabolic diseases in which redox dysregulation and mitochondrial dysfunction are better characterized [14,36]. While mechanistically plausible, direct validation in peri-implant tissues is still required. Additional challenges include lack of standardization in biomarker assessment, variability in sampling protocols, and incomplete characterization of corrosion processes under inflammatory conditions [12,13].

Despite these limitations, convergence of evidence from redox biology, mitochondrial physiology, and biomaterial science supports the concept that oxidative dysregulation represents a central mechanistic axis in peri-implant inflammation. The RAI model provides a systems-level framework that links molecular, cellular, and material-dependent processes into a unified pathogenic network. Importantly, this framework extends beyond peri-implant disease and may have broader implications for understanding redox-driven host–material interactions across biomedical implant systems. Future research should prioritize longitudinal clinical studies integrating dynamic redox biomarker profiling with disease progression, as well as experimental models directly coupling biomaterial electrochemistry with immune and mitochondrial signaling. Such interdisciplinary approaches will be essential to validate and refine the RAI model and to translate redox biology into clinically actionable strategies.

## 10. Materials and Methods

This study was conducted as a hypothesis-driven narrative review aimed at integrating current knowledge into a unified mechanistic framework describing redox amplification at the biomaterial–host interface. A structured literature search was performed using PubMed, Scopus, and Web of Science databases, covering studies published up to 2025. Search terms included combinations of “peri-implantitis”, “oxidative stress”, “mitochondrial dysfunction”, “reactive oxygen species”, “biomaterial corrosion”, and “redox signaling”. Study selection was guided by mechanistic relevance to the proposed redox amplification interface (RAI) framework. Priority was given to experimental, translational, and clinically oriented studies providing direct evidence linking immune-derived ROS, mitochondrial dysfunction, and biomaterial electrochemical processes. Additional emphasis was placed on studies elucidating redox-regulated signaling pathways, mitochondrial stress responses, and material-dependent oxidative interactions. Given the objective of hypothesis development and systems-level integration, formal systematic review criteria were not applied. Instead, an iterative selection strategy was employed to identify conceptually and mechanistically informative studies across disciplines, including redox biology, immunometabolism, and biomaterials science. This approach enabled the construction of a coherent framework while preserving biological plausibility and translational relevance. The literature search was iteratively updated to incorporate recent advances relevant to the evolving RAI model. Where appropriate, evidence from related chronic inflammatory and biomaterial-associated conditions was incorporated to support generalizability of the proposed framework.

## 11. Conclusions

Peri-implant inflammatory disease cannot be fully explained as a purely biofilm-driven condition. The RAI framework supports a model in which immune activation, mitochondrial dysfunction, and biomaterial electrochemistry form a coupled system sustaining oxidative stress at the host–implant interface. Within this framework, impaired antioxidant buffering and increased mitochondrial susceptibility represent critical transition points enabling self-reinforcing inflammatory amplification. In addition, biomaterial surfaces act as active modulators of local redox microenvironments, contributing to oxidative inputs that interact with host-derived mechanisms. Recognition of redox dysregulation as a central driver of disease provides a basis for biomarker-guided risk stratification and targeted therapeutic strategies. Modulation of immune-derived ROS, restoration of antioxidant capacity, mitochondrial protection, and optimization of biomaterial properties represent complementary approaches to improving clinical outcomes. Future studies integrating redox biomarkers, mitochondrial function, and material-specific parameters will be essential to validate this framework and support its translation into clinical practice.

## Figures and Tables

**Table 1:** Physiological versus pathological redox states at the peri-implant interface.

Biological Domain	Physiological ROS Signaling	Redox Dysregulation Under Chronic Inflammation	Functional Outcome
Innate immunity	Transient NOX2/NOX4 activation with spatial confinement	Sustained NOX activity and mitochondrial ROS amplification	Persistent inflammatory signaling
Mitochondrial metabolism	Controlled ROS supporting bioenergetic regulation	ETC complex damage and ROS-induced ROS release	Metabolic dysfunction
Osteoblast biology	ROS-mediated activation of Runx2 and Wnt/β-catenin	Oxidative suppression of osteogenic transcription	Impaired bone formation
Osteoclast differentiation	Controlled ROS facilitation of RANKL signaling	Excessive NFATc1 activation and ROS amplification	Accelerated bone resorption
Angiogenesis	Redox-regulated HIF signaling and vascular adaptation	Oxidative endothelial injury	Impaired vascular adaptation
Antioxidant defense	Nrf2-regulated glutathione homeostasis	Nrf2 exhaustion and thiol depletion	Loss of redox buffering

**Table 2:** Mechanistic layers of redox buffering collapse within the RAI model.

Redox Control System	Primary Disruption	Molecular Consequence	Pathogenic Amplification
Nrf2-Keap1 signaling	Chronic inflammatory suppression	Reduced antioxidant gene expression	Progressive ROS accumulation
Glutathione system	Excess oxidant neutralization	Shift toward oxidized cellular redox potential	Mitochondrial vulnerability
Mitochondrial integrity	ETC complex I/III oxidative injury	Increased electron leakage	ROS-induced ROS release
Mitophagy pathways	Oxidative inhibition of quality control	Accumulation of dysfunctional mitochondria	Sustained oxidative flux
NADPH regeneration	Metabolic and inflammatory stress	Impaired glutathione recycling	Failure of redox recovery

**Table 3:** Oxidative biomarkers as functional readouts of the redox amplification interface (RAI) model.

Biomarker	Biological Target	RAI Layer Represented	Dominant Redox Mechanism	Translational Relevance
8-hydroxy-2′-deoxyguanosine (8-OHdG)	Nuclear and mitochondrial DNA	Mitochondrial amplification	ROS-induced ROS release and mtDNA oxidation	Indicator of mitochondrial dysfunction and disease severity
Malondialdehyde (MDA)	Membrane lipids	Oxidative propagation layer	Lipid peroxidation and radical chain reactions	Reflects intensity of oxidative damage
Nitrotyrosine	Cellular proteins	Inflammatory redox coupling	Peroxynitrite-mediated nitrative stress	Marker of chronic inflammatory activation
Total antioxidant capacity	Systemic and local redox reserve	System-level resilience	Antioxidant network exhaustion	Predictor of disease susceptibility
Protein carbonyls	Structural and enzymatic proteins	Downstream oxidative damage	Irreversible protein oxidation	Indicator of cumulative oxidative injury
GSH/GSSG ratio	Cellular thiol pool	Antioxidant buffering layer	Redox buffering depletion	Marker of oxidative vulnerability
Biomarker	Biological target	RAI layer represented	Dominant redox mechanism	Translational relevance

## Data Availability

No new data were created or analyzed in this study. Data sharing is not applicable to this article.
